# Up-to-date quality survey and evaluation of neonatal screening programs in China

**DOI:** 10.1186/s12887-024-04528-1

**Published:** 2024-01-20

**Authors:** Jinming Zhang, Lizi Jin, Penghui Feng, Yu Fei, Wen Li, Ting Jiang, Zehao Zhang, Falin He

**Affiliations:** 1 Present Address: National Center for Clinical Laboratories, Institute of Geriatric Medicine, Chinese Academy of Medical Sciences, Beijing Hospital/National Center of Gerontology, Beijing, China; 2 Chinese Academy of Medical Sciences and Peking Union Medical College, Beijing, China; 3 Present Address: Department of Obstetrics and Gynecology, National Clinical Research Center for Obstetric & Gynecologic Diseases, Peking Union Medical College Hospital, Beijing, China; 4 West China School of Public Health, West China Fourth Hospital, Sichuan University, Chengdu, China

**Keywords:** Neonatal screening, Quality management, Quality performance, Quality evaluation

## Abstract

**Aims:**

To thoroughly evaluate the quality of the entire process of neonatal screening (NBS) in China.

**Methods:**

We collected survey questionnaires from 54.4% (135/248) of NBS institutions in China and conducted on-site visits to 20 of these facilities to validate the data. The quality performance of the institutions was evaluated, and differences across various factors were analysed.

**Results:**

Merely 62.5% of the provinces had acceptable performance in neonatal screening. Institutions with limited staff were more prone to organizational management shortcomings. Institutions in provinces with a per capita GDP below 10,000 USD exhibited lower quality control levels than those with a per capita GDP between 10,000 and 15,000 USD. Obstetrics departments have a lower awareness of quality control compared to other blood collection facilities.

**Conclusions:**

A nationwide, comprehensive quality control system for continuous enhancements in quality management, screening, diagnosis, and treatment is imperative to ensure prompt diagnosis and intervention.

**Supplementary Information:**

The online version contains supplementary material available at 10.1186/s12887-024-04528-1.

## Introduction

Neonatal screening (NBS) [1] for inherited metabolic disorders (IMDs) [2] is a systematic health care service designed to prevent adverse outcomes in an infant’s early life [3]. NBS involves a highly complex system of processes, including health education, specimen collection and delivery, laboratory screening and testing, and post-laboratory management, across multiple medical institutions and personnel [4, 5]. Ensuring the quality and accuracy of screening results is essential, as false-positive or false-negative results may lead to devastating consequences for affected individuals and their families [6–9]. Consequently, regular evaluation and monitoring of NBS programs are required to identify areas for improvement and uphold the highest standards of care. [1]

Several studies have assessed the quality performance of NBS programs across various countries. Loeber et al. (2021) [10] provided a comprehensive overview of the new technologies, policies, and data regarding newborn screening in the European region, from 2010 to 2020. They also identified future development directions and called for international collaboration. Lüders A et al. [11, 12] (2021) evaluated the German NBS system using “DGNS reports” and found satisfactory regulated components but a flawed tracking system. The American NewSTEPs 360 project (2020) [9], aiming to reduce reporting and intervention turnaround times, employed eight QIs, revealing good performance in participating NBS programs from sample collection to results reporting. Padilla CD et al. [13] (2020) reported the successful implementation of the Philippine Performance Evaluation and Assessment Scheme (PPEAS) in the Philippines. It proposed improvement measures based on their analysis of neonatal screening in developing countries, along with their personal experience in the Philippines.

However, NBS quality performance in China is typically evaluated via external quality assessment (EQA) measuring laboratory metabolic production [14]. These EQA schemes only focus on the laboratory analytical process, overlooking quality assurance for services beyond the laboratory. CW Yu et al. [15] (2021) established 16 QIs covering the entire screening process to assess and monitor the quality performance of NBS programs in Southeast China from 2015 to 2019, showing that the quality of the NBS in Southeast China improved continuously. Nevertheless, there is currently a lack of nationwide surveys on full-process newborn screening in China, and the comprehensive quality of the entire NBS system remains unclear.

This study examines and measures the current practice and quality performance of Chinese NBS programs. To identify strengths and weaknesses in current NBS programs, we measured the quality levels of individual components. The factors contributing to poor/good quality/practice were examined through subgroup analysis. This study aims to provide a scientific basis for the future development of more advanced NBS programs by reviewing the current situation of NBS programs in China.

## Materials and methods

### Survey subjects

Qualified NBS institutions in China. All respondents voluntarily submitted their data. The specifications of the Chinese NBS program involved in this study are illustrated in Supplementary Material.

### The designated NBS program in China

The components and the required processes of the Chinese NBS program are illustrated in Supplementary Table 1. The NBS center, along with its affiliated blood collection agencies, comprises the NBS service agencies in China, which are responsible for promoting awareness and education regarding the screening of IMDs. The NBS program includes health education and publicity, specimen collection and delivery, laboratory screening and testing, and clinical diagnosis and treatment.

### Survey

A survey consisting of 99 questions, which address comprehensive and key aspects of the NBS system, is presented in Supplementary Tables 1 and Supplementary Table 2. These questions include both qualitative and quantitative inquiries.

The initial questionnaire is based on the Program Evaluation and Assessment Scheme (PEAS) from the United States, the assessment regulations of the Chinese Newborn Screening (NBS) management department for NBS Centers, and our previous research(It mainly involves using Quality Indicators (QIs) to construct the Newborn Screening (NBS) quality system and questionnaire design) [16]. At this stage, the questionnaire consists of numerous questions but does not include detailed scoring criteria. To adapt to the characteristics of an online survey and China’s actual condition, we meticulously selected and refined a subset of questions. Afterward, we conducted three Delphi rounds involving 20 experts (5 blood collection specialists, 5 medical laboratory experts, and 10 clinical doctors) to further refine the questionnaire indicators. These experts engaged in discussions and made necessary modifications to enhance the feasibility of the plan, consequently providing specific scoring criteria. Subsequently, two experts with senior professional titles from the National Office for Maternal and Child Health Surveillance of China/National Center for Birth Defect Surveillance of China, along with an expert from Zhejiang University Children’s Hospital, reviewed the scoring of all questions and their corresponding criteria. The details of the survey questions are presented in Supplementary Table 2.

The survey was fielded on the official website of the National Center for Clinical Laboratories (https://www.nccl.org.cn/mainEn). As all NBS Centers are obligated to participate in the EQA program, we extend invitations to them through the EQA webpage. Participants voluntarily choose to participate in any of the projects and filled out a structured survey online. The participating institutions completed their relevant part of the survey according to their roles in NBS health care. Supplementary Fig. 1 shows the NBS program in China.

### Quality control

All participants underwent comprehensive online training to receive explanations for each indicator before answering the questionnaire. During this session, we addressed any questions or concerns raised by the participants and guided preparing and verifying relevant materials before entering data. Before completing the questionnaire, we conducted a random follow-up survey by resending questions to participants and comparing responses from the initial questionnaire. In cases where discrepancies or errors were identified, we contacted the respective participants via phone to confirm and clarify their responses. After completing the questionnaires, we selected 20 institutions randomly from various regions and visited them to verify the data, out of a total of 135 institutions. The on-site verification process ensured the accuracy and reliability of the data collected through the questionnaires.

### Data analysis

Data analysis was performed by Microsoft Excel 2016 software (Microsoft Inc, Redmond, Washington, DC, USA) and R programming language (R Foundation for Statistical Computing, Vienna, Austria). We scored each answer based on the scoring criteria to quantify quality of performance. The full scores of subsection-organizational managements, screening management, diagnosis and treatment management, and quality management for NBS sample collection agencies were 99 points, 393 points, 508 points, and 300 points, respectively.

We displayed maximum, mean, and minimum quality performance scores in different provinces and applied quartiles to describe each subsection’s performance distribution. The normality test was conducted using the Shapiro-Wilk test. The Mann-Whitney U test was performed on two independent non-normal data sets, while the Kruskal-Wallis rank sum test was applied to three separate non-normal data sets. We considered scores within 95% of the total score excellent, within 90% of the total score as satisfactory, within 80% of the total score acceptable, and outside 80% of the total score as failing. P-value < 0.05 suggested the difference was statistically significant.

## Results

### Survey participants

One hundred thirty-five qualified NBS institutions from 26 provinces of mainland China participated in the survey. The number of eligible NBS institutions in 2022 China was 248, so the response rate was about 54.4%. One hundred thirty-five surveys were included in the data analysis, one hundred twenty-seven institutions were public, and eight institutions were private. Fifty-six institutions were Grade III Level A hospitals, 15 were Grade III Level B hospitals, 20 were Grade II Level A hospitals, and 21 were Grade II Level B hospitals. The remaining 23 hospitals were not rated. (In China, the government categorizes hospitals into five levels based on their size and assessment scores, with a general ranking hierarchy of IIIA > IIIB > IIA > IIB > I. Some private hospitals and grassroots clinics may not participate in this rating system).

### Quality of NBS program in participants

#### Quality of organizational management

**Fig. 1:**
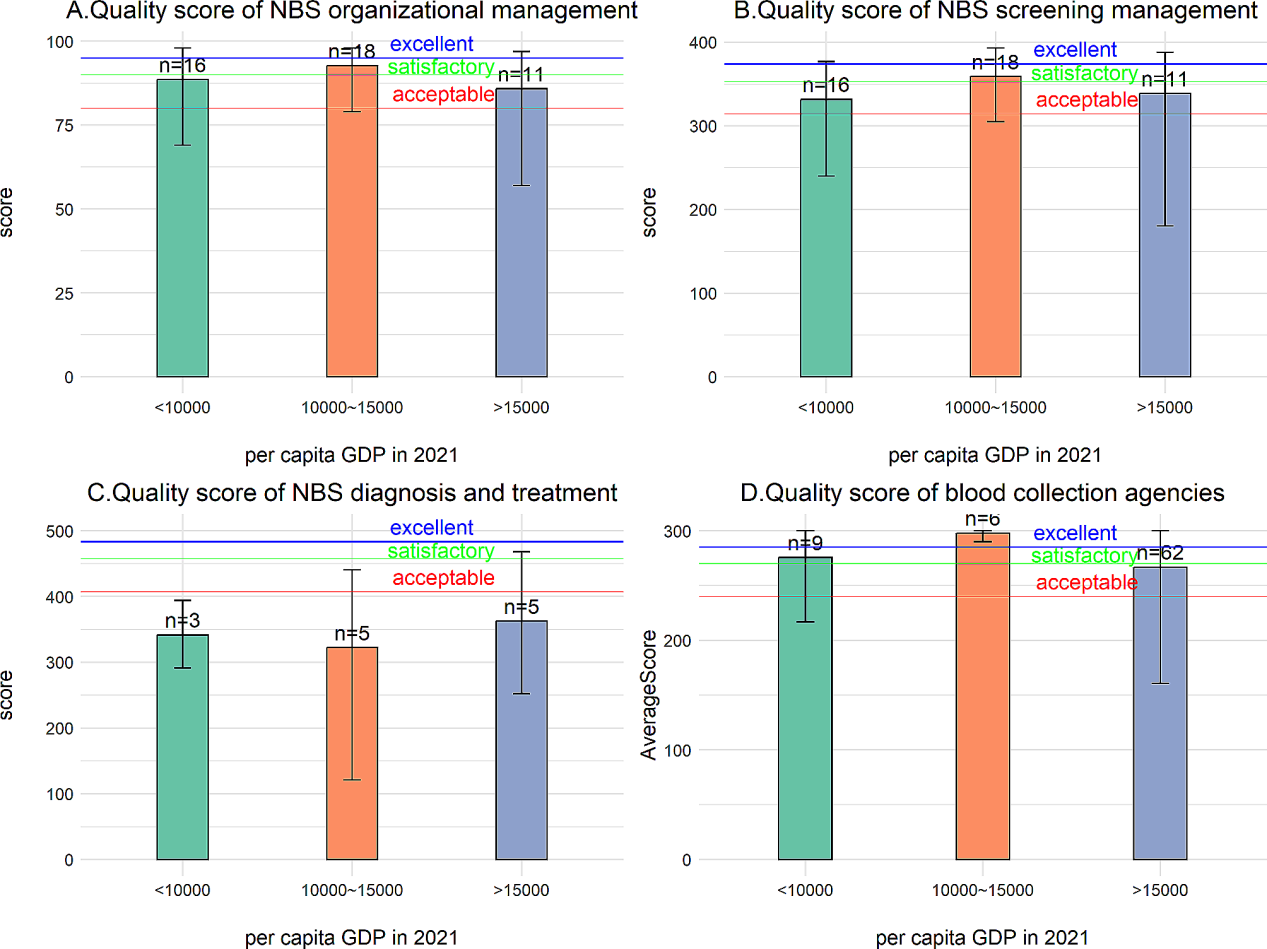
Regional Assessment of Newborn Screening Levels in China. Categorized by per capita GDP, NBS Centers are divided into three groups: <10,000 indicates provinces with per capita GDP below $10,000 USD, and so on. The bar chart shows average scores, where n = X represents the number of NBS Centers. The upper limit of the “工” shape indicates the highest score, while the lower limit represents the lowest score. From top to bottom, the three lines represent “excellent,” “satisfactory,” and “acceptable.” If below the third line, it is considered “failing”

Forty-five institutions from 25 provinces participated in this section. According to our evaluation criteria, the performance of 37.8% of institutions (17/45) is excellent, 22.2% (10/45) is satisfactory, 28.9% (13/45) is acceptable, and 11.1% (5/45) is failing. Divide NBS Centers into three categories based on the GDP ranking of the provinces they are located in. Calculate the average score, highest score, and lowest score (Fig. 1A). Table 1 displays the 25th, 50th, and 75th percentiles of scores for each subcategory in organizational management. Table 2A shows a statistically significant difference (*p* < 0.05) in the management level among institutions with different numbers of staff, indicating that institutions with fewer staff may be deficient in management level. Other factors, including area, department category, economic situation, and hospital level, show no statistically significant difference (*p* > 0.05).

**Table 1:** The distribution of the quality magnitude of newborn screening programmes in participating institutions. * For details of specifications for each subsection of NBS program, please see the Supple Material

Sections	Ideal total scores		Scores	
Subsections*			Percentages, %	
		25th	50th	75th
**Organizational Management**	**99**	**88**	**92**	**95**
Institutional setting	22	19.5	22	22
Personnel	26	20	25	25.5
Laboratory construction	27	23	26	26
Rules construction	16	15	16	16
Information system construction	8	5	7	7
**Screening Management**	**393**	**331**	**353**	**371**
νPrescreening health education	3	3	3	3
νPretesting quality control	65	52	63	65
νTesting quality control	118	109	118	118
νPost-testing quality control	188	141	155	174.5
νFollow up	19	19	19	19
**Diagnosis and Treatment**	**508**	**302.5**	**339**	**413**
νCase diagnosis	128	64.5	78	108
νTreatment and effect	360	187	252	283
νMedical record management	20	20	20	20
**Quality of Blood Collection Agencies**	**300**	**258**	**282**	**300**
υPersonnel	15	15	15	15
υInstitution construction	30	30	30	30
υPublicity and health education	35	35	35	35
υBlood collection	130	112.5	130	130
υQuality of specimens	80	42	80	80
υArchives preservation	10	8	10	10

* For details of specifications for each subsection of NBS program, please see the Supple Material

**Table 2:** Difference analysis in quality of NBS programmes of participating institution

**2A. analysis of differences in organizational management**					
**Variables**	**Number of hospitals**	**Median (IQR)**	**Shapiro-Wilk test** ***P*** **value**	**Testing methods**	***P***
**Hospital level**					
Level III Class A	26	94(89.25 ~ 95.75)	< 0.001	Mann-Whitney U test	0.081
Others	19	89(84 ~ 94.5)	0.010		
**Economic situation (per capita GDP of each province in 2021)**					
> 15,000 USD	11	95(79 ~ 95.5)	< 0.001	Kruskal-Wallis rank sum test	0.092
10,000 ~ 15,000 USD	18	95(89.25 ~ 96.25)	0.008		
< 10,000 USD	16	89(87.5 ~ 92.25)	0.015		
**Department category**					
NBS center	11	95(79 ~ 95.5)	0.080	Kruskal-Wallis rank sum test	0.890
Clinical Laboratory	18	94(89.25 ~ 95)	< 0.001		
Others	13	92(89 ~ 95)	< 0.001		
**Number of staff in the department**					
< 10	11	86(74 ~ 90.5)	0.166	Mann-Whitney U test	0.014
> 10	34	94(89 ~ 95.75)	< 0.001		
**Area difference**					
Western China	16	92(89 ~ 94.25)	< 0.001	Kruskal-Wallis rank sum test	0.678
Central &Northeast China	14	90(88 ~ 95)	0.471		
Eastern China	15	95(87 ~ 96)	0.002		
**2B. analysis of differences in quality control**					
**Variables**	**Number of hospitals**	**Median (IQR)**	**Shapiro-Wilk test** ***P*** **value**	**Testing methods**	***P***
**Hospital level**					
Level III Class A	26	357.25(334.25 ~ 374.75)	0.007	Mann-Whitney U test	0.132
Others	19	334(326 ~ 363)	< 0.001		
**Economic situation (per capita GDP of each province in 2021)**					
> 15,000 USD	11	364(333 ~ 376)	0.002	Kruskal-Wallis rank sum test	0.028
10,000 ~ 15,000 USD	18	360(352.75 ~ 374.88)	0.329		
< 10,000 USD	16	333.5(322.5 ~ 341.75)	0.030		
**Department category**					
NBS center	11	339.5(325 ~ 369.13)	0.118	Kruskal-Wallis rank sum test	0.414
Clinical Laboratory	18	360(341.5 ~ 366.75)	0.165		
Others	13	334(323 ~ 371)	0.021		
**Number of staff in the department**					
< 10	11	353(323 ~ 363)	0.165	Mann-Whitney U test	0.484
> 10	34	353.5(332.25 ~ 371.38)	0.011		
**Area difference**					
Western China	16	334(329.25 ~ 360)	0.609	Kruskal-Wallis rank sum test	0.489
Central &Northeast China	13	352(329 ~ 377)	0.031		
Eastern China	16	358(341 ~ 368)	< 0.001		
**2C. analysis of differences in performance differences of blood collection agencies**					
**Variables**	**Number of hospitals**	**Median (IQR)**	**Shapiro-Wilk test** ***P*** **value**	**Testing methods**	***P***
**Hospital level**					
Level III Class	28	287.5(262.5 ~ 300)	< 0.001	Kruskal-Wallis rank sum test	0.177
Level II Class	31	265(235.5 ~ 298.6)	0.119		
Others	18	287.5(259.25 ~ 299.5)	0.002		
**Department category**					
Obstetrics	60	269.5(248.25 ~ 299.4)	< 0.001	Mann-Whitney U test	0.030
Others	17	297(281.35 ~ 300)	0.001		
**Number of staff in the department**					
< 100	24	284.18(257 ~ 300)	0.002	Mann-Whitney U test	0.861
> 100	52	282.5(259.5 ~ 298.3)	< 0.001		

NBS Centers are grouped based on various criteria, and a differential analysis is conducted on the scores across these different groups. “Median (IQR)” represents the median and interquartile range of scores for each group. The “Shapiro-Wilk test P value” displays the p-value of the normality test. “Testing methods” refer to the methods used for assessing differences between groups. “P” denotes the p-value of the test for differences

#### Quality of screening

The 45 institutions participating in this section come from 25 provinces. According to our evaluation criteria, 22.2% of institutions (10/45) are excellent, 28.9% (13/45) satisfactory, 20.0% (9/45) acceptable, and 28.9% (13/45) failing. Divide NBS Centers into three categories based on the GDP ranking of the provinces they are located in. Calculate the average score, highest score, and lowest score (Fig. 1B). Table 1 displays the 25th, 50th, and 75th percentiles of scores for each subcategory in screening management. Table 2B shows a statistically significant difference (*p* < 0.05) in quality control levels among institutions with different economic situations. Further Dunn test with Bonferroni correction for pairwise comparisons shows a statistically significant difference (*p* < 0.05) between the group with income levels of 10,000 ~ 15,000 USD and those with income levels less than 10,000 USD. In contrast, no statistically significant differences were found in other pairwise comparisons. Other factors, including area, department category, staff numbers, and hospital level, show no statistically significant difference(*p* > 0.05).

#### Quality of diagnosis and treatment

The 13 institutions participating in this section come from eleven provinces. According to our evaluation criteria, none of the institutions is excellent, 7.7% (1/13) satisfactory, 15.9% (2/13) acceptable, and 76.9% (10/13) failing. Divide NBS Centers into three categories based on the GDP ranking of the provinces they are located in. Calculate the average score, highest score, and lowest score (Fig. 1C). Table 1 displays the 25th, 50th, and 75th percentiles of scores for each subcategory in Diagnosis and Treatment. Due to the small sample size, we did not perform a differential test for the Quality of Diagnosis and Treatment. According to our grading criteria, this part of the performance is not quite good (Fig. 1C).

#### Quality of NBS blood collection agencies

The 77 institutions participating in this section come from 10 provinces. According to our evaluation criteria, the quality of 46.8% (36/77) institutions were excellent, 10.4% (8/77) was satisfactory, 26.0% (20/77) were acceptable, and 16.9% (13/77) were failing. Divide NBS Centers into three categories based on the GDP ranking of the provinces they are located in. Calculate the average score, highest score, and lowest score (Fig. 1D). Table 1 displays the 25th, 50th, and 75th percentiles of scores for each subcategory in Quality of Blood Collection. Table 2C shows that the Department category had a statistically significant difference in the quality performance of NBS sample collection agencies (*p* < 0.05), indicating that the blood collection level in obstetrics may be weaker than others. Hospital level and number of staff show no statistically significant difference(*p* > 0.05).

#### Quality performance of the whole NBS programs

**Fig. 2:**
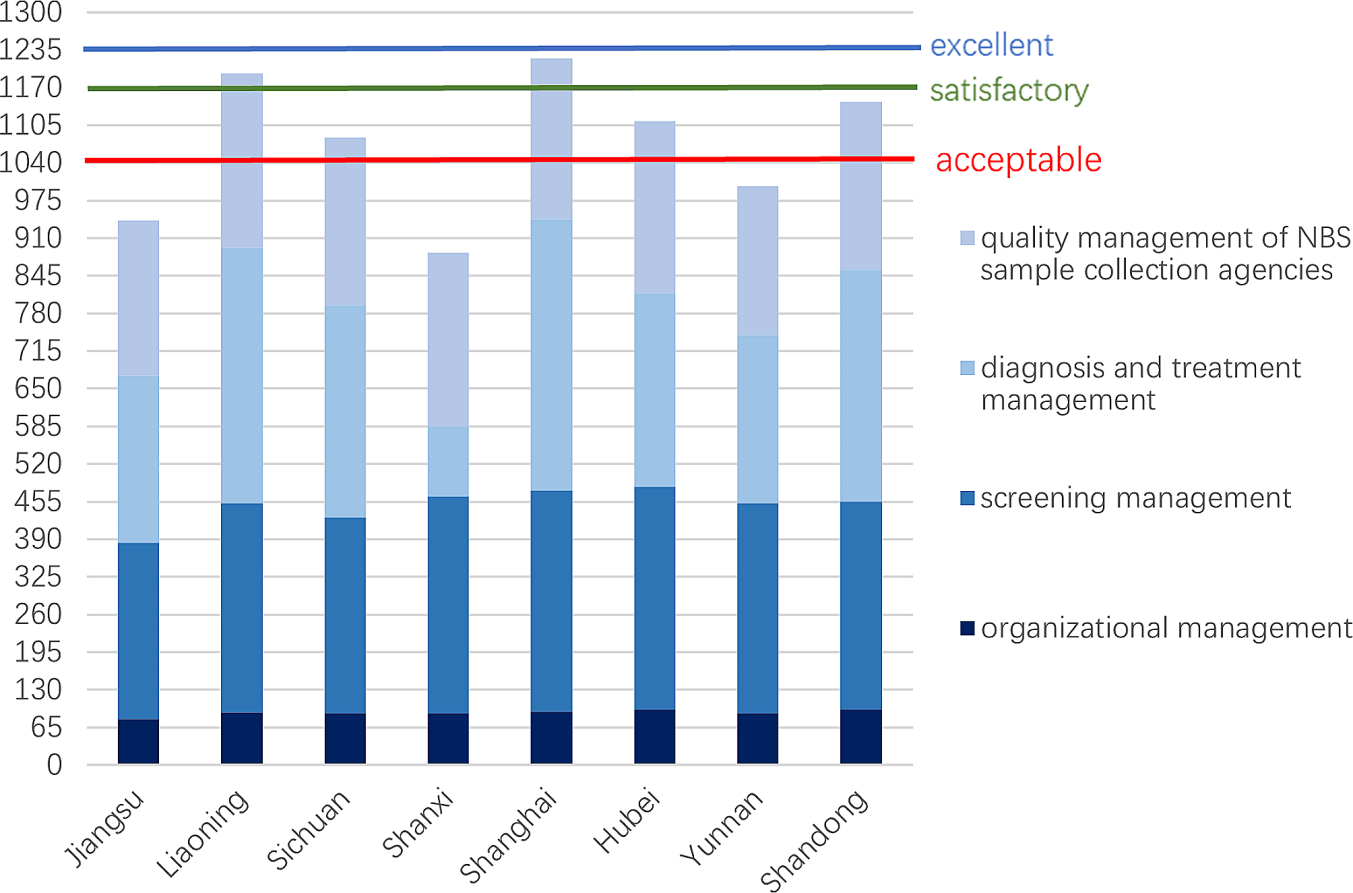
Quality performance of the whole neonatal screening system in eight provinces of China. From top to bottom, the three lines represent “excellent,” “satisfactory,” and “acceptable.” If below the third line, it is considered “failing”

Since participation in the projects is voluntary, an organization typically does not participate in all of them. Therefore, we choose to sum up the average scores of four projects from each province for the overall evaluation (Fig. 2). No province had excellent performance; 25.0% of participating provinces had satisfactory performance, 37.5% had acceptable performance, and 37.5% had failing performance. The quality of areas of higher-than-average GDP (1104.5 points) was slightly higher than those of lower-than-average GDP (1040.3 points). The quality of the coastal regions (1125.1 points) was also slightly higher than midwestern areas (1019.7 points).

## Discussion

Our investigation revealed that there is a relatively small disparity between different regions and different levels of hospitals in China, but some aspects still require improvement. We observed that smaller organizations with limited staff often exhibit deficiencies in organizational management. Consequently, it is crucial to focus on enhancing management standards in these units, as their size may lead to inadequate attention to formal organization and administration.

Additionally, we identified that obstetrics departments have a lower awareness of quality control compared to other blood collection facilities. NBS is a multidimensional and systematic healthcare process involving multiple institutions and diverse personnel, such as midwives, nurses, laboratorians, paediatricians, and maternal and child healthcare workers. This intricate process highlights the importance of effective communication and collaboration among all stakeholders.

Interestingly, our findings show that institutions in provinces with a per capita GDP below 10,000 USD had lower quality control levels than those with a per capita GDP between 10,000 and 15,000 USD. However, no discernible gap was observed when compared to provinces with a per capita GDP above 15,000 USD. This phenomenon may be due to the wealthiest provinces having already expanded their NBS services to grassroots institutions.

China has invested substantial financial funds in neonatal screening and legislated it in the 1990s. By 2030, newborns’ screening rate for genetic and metabolic diseases will reach 98% [17]. To this end, governments actively carry out free newborn screening. Welfare lottery and various foundations have also generously funded the treatment of screening-positive children. The screening rates in many provinces have already exceeded 99%. In terms of screening coverage, China has performed relatively well, not inferior to the United States (97%, within 24-48 h) [9], UK(99%, within 3–4 days) [10, 18]. The blood sampling time in China (within 3–7 days) is relatively later compared to Europe (the median sampling window of 48–72 h) [10] and the United States, but this may be because China primarily conducts screening for phenylketonuria (PKU) and congenital hypothyroidism (CH).It is generally believed that PKU should be tested after 12 h postpartum [19], and CH is recommended to be screened for between four to six days [20]. Testing only for PKU and CH appears relatively restricted when compared to developed countries such as the America (at least 29 of the 35 core disorders) [21, 22], Germany [17, 23], and England [9, 24]. However, this discrepancy could be attributed to regional variations in the prevalence of specific diseases, as well as financial factors.

Despite the high screening rate in China, it is essential to recognize that Screening is different from diagnostics [25]. Our study revealed suboptimal overall performance and diagnostic and treatment levels in Chinese neonatal screening institutions, with a significant proportion failing to meet our standards. After reviewing the questionnaire, the primary deficiencies identified include inadequate timeliness in diagnosis, treatment, and long-term tracking. Accelerating the speed of diagnosis and treatment could significantly improve the outcomes of IMDs. Many countries also have trouble organising short term and long term follow up. In this regard, the NewSTEPs 360 program in the United States and the tracking system in Germany can serve as valuable references. NewSTEPs 360 aided NBS programs in tackling pre-analytical and analytical phase challenges. Strategies for accelerating diagnosis could include enhancing preconception and prenatal education (This is China’s biggest deficiency at present), transportation optimization, and extended laboratory operating hours [9]. Lüders A et al. [10] reported that a lack of a tracking system resulted in treatment delays for 12% of children with hypothyroidism, leading to approximately 54,000 children (around 20%, including 10% of initially screening-positive children) being lost to follow-up. In contrast, the implementation of a tracking system in Bavaria, Germany, reduced the loss to follow-up rate from 57 to 1% [26]. However, replacing phone follow-ups with mobile apps or social media could better suit Chinese habits. However, due to the high volume of advertisement calls, Chinese people are not accustomed to answering unfamiliar phone calls. Utilizing popular mobile applications for automated notifications may be more welcomed and can help reduce costs and long-term loss to follow-up rates.

Currently, newborn screening in China is jointly managed by healthcare institutions at all levels. There are variations in policies implemented across different regions, and the scientific validity of these policies is questionable. In the face of this chaos, this article calls for nationwide unified management of newborn screening. Firstly, an institution needs to be established to implement a full-process quality control that extends beyond laboratory controls. This entity could be responsible for the collection and analysis of nationwide data and strengthening international cooperation. However, the most critical task is to unify evaluation standards, comprehensively assess the screening, diagnostic, treatment and quality control levels of NBS Center, and thus promote comprehensive and continuous improvement to maximize child interests.

Furthermore, drawing inspiration from the Expert Opinion of the European Union(The European Union and China share similarities in certain situations), it is suggested to form an expert network and develop a decision-making matrix consisting of representatives from various regions [25, 27–29]. Given that the majority of IMDs are rare diseases, it is crucial that all decisions, including the aforementioned evaluation standards and the promotion of international cooperation, should be implemented only after discussion by relevant experts, especially health economists. At present, the nationwide mandatory screening for IMDs in China only includes PKU and CH. The addition of screening for locally prevalent genetic diseases can be discussed through a decision-making matrix that considers the characteristics of each region. Meanwhile, population-based management can also be determined using the same decision-making matrix. These two institutions should operate in parallel and cooperate with each other.

The primary constraint of our study is the relatively small sample size, with surveys conducted only across 135 NBS institutions. However, this is mainly due to the overall limited number of NBS institutions in China, which is only 248. There are only thirteen institutions involved in the diagnosis and treatment part. This may be because we sent out invitations through the EQA system, the majority of those who received invitations were laboratory personnel. In China, laboratories are often relatively separate from treatment institutions. Additionally, most blood collection agencies come from Jiangsu province. This may be because Jiangsu, as the province with the highest per capita GDP, has a higher number of blood collection agencies. Despite these constraints, our study remains highly representative, offering a comprehensive portrait of the current state of NBS in China.

### Electronic supplementary material

Below is the link to the electronic supplementary material.

Supplementary Material 1

Supplementary Material 2

Supplementary Material 3

Supplementary Material 4

## Data Availability

The data that support the findings of this study are available from the corresponding author, upon reasonable request.
