# Supplementary material for: Up-to-date quality survey and evaluation of neonatal screening programs in China
Source: BMC Pediatr. 2024 Jan 20;24:65. doi: 10.1186/s12887-024-04528-1 (PMC10799474; doi:10.1186/s12887-024-04528-1)
Supplement: Supplementary file 1 — Supplementary Material 1 [file 12887_2024_4528_MOESM1_ESM.pdf]

Suppl Material Introduction of specifications for Chinese newborn screening agencies involved in this study.

Newborn screening (NBS) agencies in China are required to follow:

(I) Requirements in Institutional Setting and Management

1. Medical institutions that carry out newborn screening (NBS centers) must have obtained approvals from the health administration of the provinces, autonomous regions, and municipalities directly under the central government, and the annual screening volume should exceed 30,000.

2. NBS centers should perform quality control and training regularly for their signed blood collection agencies.

3. Medical institutions that carry out screening and diagnosis should set up special clinics for clinical diagnosis, or designate specialists who are responsible for the diagnosis, treatment and management of inherited metabolic diseases (IMDs).

(II) Requirements in Personnel

1. The qualifications of the person in charge of the NBS laboratory should meet the following requirements: (1) medical-related bachelor's degree or above, (2) senior professional title, (3) experience in pediatrics or clinical laboratory work, (4) engaged in NBS work for more than 5 years, and (5) master the operation and management of the NBS service.

2. NBS centers should ensure that there is an appropriate number of laboratory technicians with the required education, training and abilities to provide NBS services, and their qualifications meet the following requirements: (1) technical secondary school education or above, (2) more than 2 years of experience in clinical laboratory work, (3) have a title of technician or above, and (4) received the relevant knowledge and skills training for NBS organized by the health administration department at or above the provincial level, and obtained the technical qualifications certificate.

3. NBS centers should ensure that clinicians have the necessary education, training and abilities to provide clinical diagnosis and treatment services that meet the needs of patients, and ensure whether the qualifications of these personnel meet the requirements: (1) must meet the qualifications of practicing physicians, (2) have intermediate or above pediatric clinical professional titles, and (3) should have knowledge of inherited metabolic diseases, more than 5 years, and (4) master the operation and management of the NBS service.

4. NBS centers should ensure that clinicians have the necessary education, training and abilities to provide clinical diagnosis and treatment services that meet the

needs of patients, and ensure whether the qualifications of these personnel meet the requirements: (1) must meet the qualifications of practicing physicians, (2) have intermediate or above pediatric clinical professional titles, and (3) should have knowledge of inherited metabolic diseases, endocrinology and other relevant knowledge and have passed NBS skills trainings.

5. Personnel engaged in NBS should have received continuing education training, e.g., participating in national and provincial training courses and obtaining training class credit certificates or participating in academic conferences and obtaining credit certificates.

(III) Requirements in Laboratory Construction

1. Laboratory Instruction Site: The laboratory site for NBS should meet the following requirements: (1) 2 laboratory rooms with a usable area of at least 40 square meters; (2) 2 comprehensive rooms with at least 20 square meters for dried blood spot (DBS) checks and acceptance, computer entry, and data registration and preservation; (3) 1 DBS storage room or cold storage room for the long-term storage of DBS samples; (4) house area should be appropriately increased according to the amount of and the types of diseases to be screened; and (5) the laboratory's working partitions should be reasonably set up, the space layout should be convenient for the experimental process, the amount of and the types of diseases to be screened; and (5) the laboratory's working partitions should be reasonably set up, the space layout should be convenient for the experimental process, the identification should be clear, and the temperature and humidity records should be available.

2. Equipment Configuration: The configuration of the experimental equipment should comply with the following requirements: (1) At least 1 microplate reader or fluorescence analyzer for experimental testing; (2) At least one plate washing instrument for washing the experimental plate; (3) At least one oscillator for mixing experimental reagents; (4) At least one computer (including printers) for data processing; (5) At least 1 thermostat or water bath for experimental thermostatic treatment; (6) At least one 2–8 °C refrigerator for reagent storage; (7) At least 2 multichannel samplers for experimental sampling; (8) At least 2 single-channel samplers for experimental sampling; (9) Puncher for punching DBS samples; (10) At least one ultraclean worktable for experimental operation of bacterial inhibition methods; and (11) General low-value laboratory supplies.

(IV) Requirements in Rules Construction

1. There should be personnel post responsibility rules and staff codes of conduct, and they should be constantly updated.
2. The diagnosis and treatment rules for IMDs should comply with the "Technical Standards for Screening Neonatal Diseases" and be continuously updated.
3. There should be referral, recall, and follow-up rules and a statistical collection and reporting rule, and they should be updated in a timely manner.

4. There should be a file management rule and an information management and security rule for confirmed patients, and they should be continuously updated.
5. There should be management rules for equipment, reagents and materials, and specimen registration and preservation.

(V) Requirements in Information System Construction

1. NBS centers should have a neonatal screening information system. The whole process, from blood collection to reporting, should be computerized and there should be an information module for preliminary screening and preliminary diagnosis.
2. NBS centers that carry out the diagnosis and treatment of inherited metabolic diseases should have an information system for the diagnosis and treatment of inherited metabolic diseases. The entire process, from rescreening and diagnosis to follow-up, should be computerized and include complete recall information.

(VI) Requirements in Informed Consent

Informed consent should meet the requirements, and the following contents should be included: (1) Name of the mother, neonatal sex, date of birth and medical record number of hospitalization; (2) Sections for popularizing newborn screening health education publicity and related policies; (3) A section for informed choice of the family member of the child, including the signature and date of the signature by the guardian; (4) If the guardian does not agree to the screening after the neonatal screening health education, the guardian should be informed of the possible adverse consequences of disease; (5) If the guardian does not agree to accept the neonatal screening, the guardian's signature, signature date, current address and contact information should be recorded; and (6) Medical (caregiver) statement section (example statement: I have informed the caregiver of the nature, purpose, risk, necessity and cost of genetic metabolic disease screening, and have answered any questions related to this examination), medical (caregiver) signature and date of the signature.

(VII) Requirements in Equipment and Reagents

Laboratory instruments, equipment, reagents and analysis software used for screening should comply with the basic requirements of the "Administrative Measures for Clinical Laboratories of Medical Institutions" (Weiyifa [2006] No. 73) and have been approved for registration or filing by the Food and Drug Administration. In addition, the equipment should be calibrated or maintained regularly.

(VIII) Requirements in Specimen

1. Completeness of specimen information: When the newborn screening laboratory receives the screening specimens, there should be a dedicated person responsible for the acceptance of the specimens, and recording the time of receipt of the specimens, the number of specimens, and the state of the specimens, with records.

2. Unqualified specimen rate: Unqualified specimens mainly include the following: (1) insufficient specimen collection, which makes it difficult to meet experimental testing and storage requirements; (2) specimens that are contaminated; (3) specimens that are not submitted for testing in an appropriate time after the specimens are collected, placed for a long time or in storage conditions that are unqualified; (4) specimen information is incomplete; and (5) other unqualified situations.

3. Recollection of unqualified specimens: For unqualified specimens, the laboratory should immediately notify the blood collection agency to retake the blood sample.

4. Specimen turnaround time before testing: the collection of the DBS sample to the receipt of the DBS sample in the laboratory should be finished within a short time (within 5 working days).

(IX) Requirements in Laboratory Testing Standard Operation Procedure (SOP)

The SOP for laboratory testing should be complete, and the operation of laboratory technicians during sample processing and testing should be consistent with the SOP documents ("laboratory testing" includes testing technology, the interpretation of results, laboratory quality control, and biosafety).

(X) Requirements in Measurement Procedures Performance

1. The laboratory should periodically verify the performance of the confirmed measurement systems.

2. Internal quality control should be performed for all NBS testing items.

3. Internal quality control should include quality control charts, analysis records or reports of the reason for the results being out of control; corrective measures should be taken and recorded after out of control.

4. The CV of NBS testing should meet the requirements.

5. The laboratory should regularly participate the external quality assessment (EQA) for NBS testing and obtain the certificate of conformity in the EQA program.

(XI) Requirements in Results Reports

1. Reports release: reports should be issued by the laboratory within 5 working days from the date of receiving qualified DBS samples.

2. The standardization of testing reports: The test report should contain the following information: the mother’s name, child’s birth date, sampling date, test date, sample number, screening result, tester and results reviewer’s signature.

3. Quality control measures for reports issuing: The process of report issuance should include quality control measures to reduce errors, such as a review of the test results, a review of sample information, etc.

(XII) Requirements in Notification

When the screening result is positive, medical staff should fulfill the obligation of notification, explain the reasons for the re-examination of children with suspected, and increase the recall rate.

(XIII) Requirements in Recall

The screening center uses various methods to immediately notify the neonatal guardian to take the screening-positive children to the screening center clinic or the designated referral unit to conduct the diagnosis test on the children in a timely manner (Note: the diagnosis test is in progress or has been completed to be considered as a recall).

(XIV) Requirements in Posttesting Process

1. The storage of testing files after testing: Laboratory testing data must be kept intact and backed up electronically or with paper data in a timely manner and kept for ten years.

2. The specimen storage: The storage of DBS specimens should meet the following requirements: the specimens should be stored at 2 ~ 8℃ (the laboratory can be stored below 0℃ if conditions permit) for at least 5 years for re-examination.

3. The standardization of medical records: All positive screening tests have a clear diagnosis, and the case writing is standardized, that is, the content of the diagnostic medical record includes the following: (1) the date of assessment of the screening results, (2) the date of diagnosis/case treatment, (3) the date of treatment/intervention (if feasible), (4) the confirmed results, and (5) the treatment results of the final case (Intervention, no intervention, follow-up disappearance).

(XV) NBS efficiency (monitoring indicators not requirements)

1. Positive predictive value of Phenylketonuria and Congenital Hypothyroidism (PKU/CH) screening testing: The percentage of the number of people who were finally diagnosed with PKU/CH and the number of people who were positively screened and recalled.
2. False negative rate of PKU/CH screening: The percentage of children who have undergone NBS but have not been successfully identified as having PKU/CH.
3. Positive (or negative) follow-up rate: The blood collection institutions should assist the screening center to do the screening and follow-up work.
4. Proportion of PKU/CH patients diagnosed at the newborn stage: The proportion of the number of PKU/CH patients who were screened and diagnosed during the neonatal period (28 days after birth) in the number of children with PKU/CH.
5. Proportion of PKU/CH patients starting treatment from the neonatal period: The proportion of children with PKU/CH who started treatment during the neonatal period in the number of children diagnosed with PKU/CH.
6. Proportion of PKU/CH patients regularly monitored Phe levels/ratios of FT4/TSH: During the treatment of children with PKU/CH, the Phe levels for PKU patients and/or FT4/TSH values for CH patients should be monitored regularly.
7. Proportion of PKU/CH patients regularly evaluated physical development status: The physical development of children with PKU/CH should be assessed regularly.
8. Proportion of PKU/CH patients regularly evaluated for mental development status: children with PKU/CH need to be assessed for their intelligence development regularly.
9. Proportion of PKU/CH patients with normal physical development status: Whether the physical development of children with PKU/CH are normal is an important indicator to measure the treatment effect.
10. Proportion of PKU/CH patients with normal mental development status: Whether the intelligence development of children with PKU are normal is an important indicator to measure the treatment effect.

(XVI) Requirements in archives management

The specialist archives and management rules should be established, and the medical records of children with PKU/CH should be established and properly managed.

***The specifications involved in this study were derived from the "Technical Standards for Screening Neonatal Diseases" issued in the website of the National Health Commission of People's Republic of China (http://en.nhc.gov.cn/).**
