# Supplementary material for: Up-to-date quality survey and evaluation of neonatal screening programs in China
Source: BMC Pediatr. 2024 Jan 20;24:65. doi: 10.1186/s12887-024-04528-1 (PMC10799474; doi:10.1186/s12887-024-04528-1)
Supplement: Supplementary file 2 — Supplementary Material 2 [file 12887_2024_4528_MOESM2_ESM.pdf]

Supplementary Table 1 The Survey structure in this study.

Part I. Organizational Management (22 questions)

- **Institutional setting and management requirements**
- **Personnel requirements ()**
- **Laboratory construction requirements**
- **Rules construction**
- **Information system construction**

Part II. Screening Management (37 questions)

- **Prescreening health education and publicity**
- **Pretesting quality control**
- **Testing quality control**
- **Post-testing quality control**
- **Follow up**

Part III. Diagnosis and Treatment Management (16 questions)

- **Case diagnosis**
- **Treatment and effect**
- **Medical record management**

Part IV. Management of Blood Collection Agencies (16 questions)

- **Personnel requirements**
- **Institution construction requirements**
- **Publicity and health education**
- **Blood collection**
- **Quality of specimens**
- **Archives preservation**
