# Supplementary figures and images for: Up-to-date quality survey and evaluation of neonatal screening programs in China
Source: BMC Pediatr. 2024 Jan 20;24:65. doi: 10.1186/s12887-024-04528-1 (PMC10799474; doi:10.1186/s12887-024-04528-1)

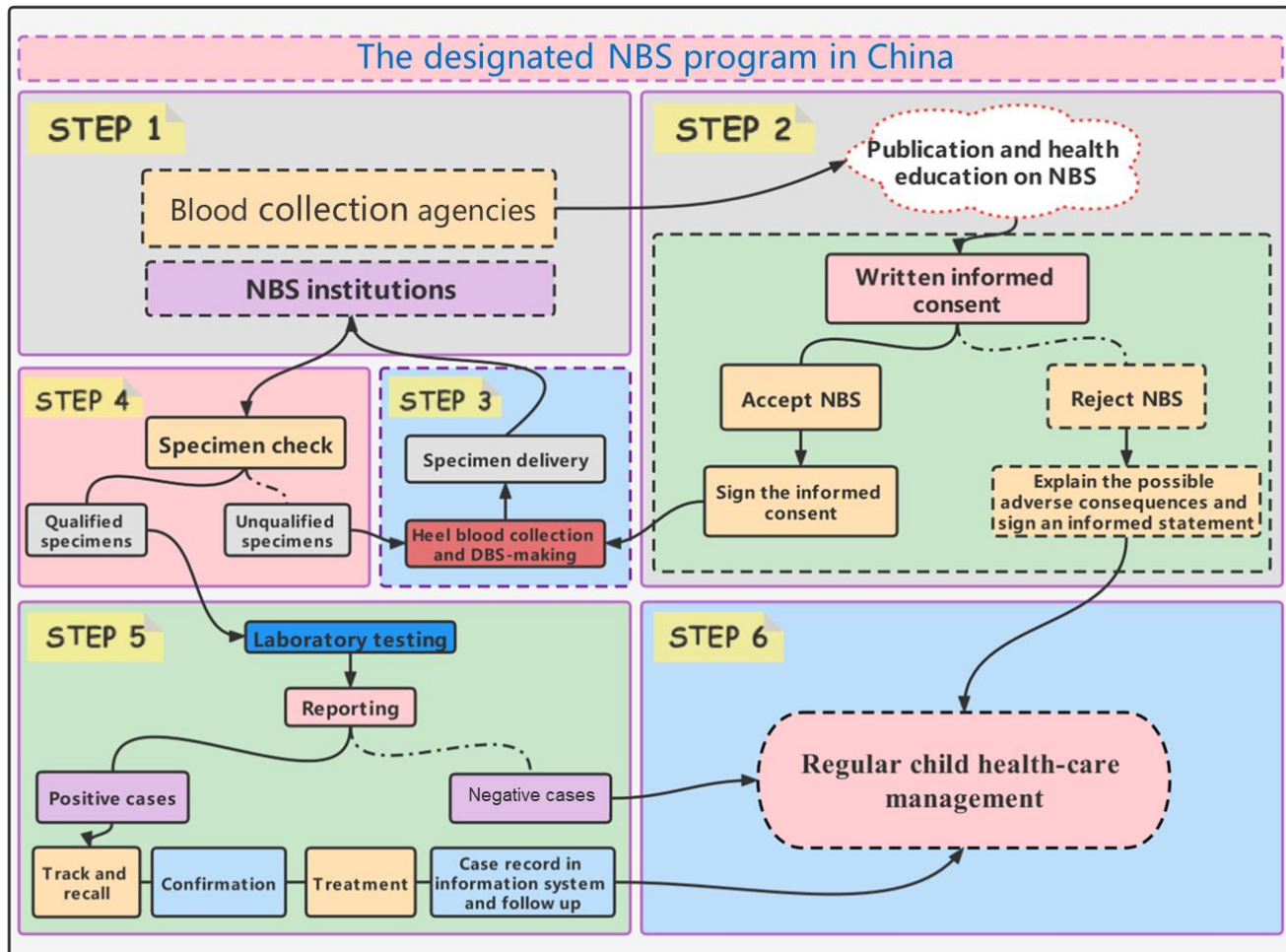

Supplementary Figure 1 The components and the required processes of the Chinese NBS program

Supplement: Supplementary file 3 — Supplementary Material 3 [file 12887_2024_4528_MOESM3_ESM.pdf]
