# Supplementary material for: Up-to-date quality survey and evaluation of neonatal screening programs in China
Source: BMC Pediatr. 2024 Jan 20;24:65. doi: 10.1186/s12887-024-04528-1 (PMC10799474; doi:10.1186/s12887-024-04528-1)
Supplement: Supplementary file 4 — Supplementary Material 4 [file 12887_2024_4528_MOESM4_ESM.pdf]

Supplementary Table 2 Details of survey questionnaires and scoring criteria.

Sections & Subsections	Questionnaires	Scoring criteria
**General information**	1. Name of your institution and province where it is located:	None.
	2. Province where your institution is located:	
	3. Your Department:	
	4. Is your institution public or private?<br>A. Public<br>B. Private	None.
	5. Level of your institution:<br>A. Grade III Level A<br>B. Grade III Level B<br>C. Grade II Level A<br>D. Grade II Level B<br>E. Others	None.
	6. Type of your institution:<br>A. General Hospital<br>B. Specialist Hospital<br>C. Maternal and Child Health Hospital<br>D. Pediatric Hospital<br>E. Commercial Clinical Laboratory<br>F. Others	None.
	7. The number of staffs in your department:<br>A. <10<br>B. 10-29<br>C. 30-49<br>D. 50-100<br>E. >100	None.
	8. Your occupation:<br>A. Laboratory leaders/technician<br>B. Clinical diagnosis and treatment doctor<br>C. Blood collector<br>D. Others	None.
**Organizational Management**		
(I) Institutional settings and management requirements	9. Did your organization obtain the health administrative approval from the health administration of the provinces, autonomous regions, and municipalities directly under the central government:<br>A. YES<br>B. NO	Chooses A can get 5 points, if chooses B, all the following questions get no point.
	10. The average annual screening amount of your institution in the past three years:<br>A. <10,000<br>B. 10,000-20,000<br>C. 20,000-30,000<br>D. >30,000	Choose D can get 5 points, and choose A/B/C will get no points.
	11. Frequency of quality assessment for the signed blood collection agencies:<br>A. Never evaluate<br>B. Once three years<br>C. Once two years<br>D. Great than or equal to once a year	Choose D can get 5 points, and choose A/B/C will get no points.

	12. Frequency of the NBS related knowledge training for blood collection agencies<br>A. No training<br>B. Once three years<br>C. Once two years<br>D. Great than or equal to once a year	Choose D can get 5 points, and choose A/B/C will get no points.
	13. Is there specialist outpatient clinics or designated specialists to diagnose and treat neonatal screening diseases:<br>A. YES<br>B. NO	Choose A to get 2 points, and choose B to get no points.
(II) Personnel requirements	14. Degree of the person in charge of the newborn screening institution:	Only Medical-related Bachelor’s Degree or above can get 1 point.
	15. Title of the person in charge of the newborn screening institution:	Only Senior Professional Title can get 1 point.
	16. Does the person in charge of the newborn screening institution have experience in pediatrics or clinical laboratory:<br>A. YES<br>B. NO	Choose A to get 4 points, and choose B to get no points.
	17. Does the person in charge of the newborn screening institution have engaged in NBS work for more than 5 years:<br>A. YES<br>B. NO	Choose A to get 5 points, and choose B to get no points.
	18. Does the person in charge of the newborn screening institution master the operation and management of the NBS service:<br>A. YES<br>B. NO	Choose A to get 5 points, and choose B to get no points.
	19. Number of laboratory technicians:___; The number of personnel whose qualifications meet the requirements:___.	At least one laboratory technician meets the requirements to get 2 points.
	20. Qualifications of the clinical diagnosis and treatment personnel meet the following qualification requirements:<br>A. Have qualifications of practicing physicians<br>B. Have intermediate or above pediatric clinical professional titles<br>C. Have knowledge of inherited metabolic diseases, endocrinology and other relevant knowledge and have passed NBS skills trainings:	Choose A to get 2 points, choose B to get 2 points, and choose C to get 2 points.
	21. Number of personnel engaged in NBS:__; Number of NBS personnel who have received at least one training:__.	All personnel engaged in the NBS must have received at least one training to get 2 points.
(III) Laboratory construction requirements	22. Which requirements does your laboratory site for NBS meet:<br>A. There're 2 laboratory rooms with a usable area of at least 40 square meters<br>B. There're 2 comprehensive rooms with at least 20 square meters for dried blood spot (DBS) check and acceptance, computer entry, and data registration and preservation<br>C. There's 1 DBS storage room or cold storage room for long-term storage of DBS<br>D. The house area can be appropriately increased according to the amount of and the types of diseases to be screened<br>E. The laboratory’s working partitions are reasonably set up<br>F. The space layout is convenient for the experiment process<br>G. The identification in the laboratory is clear	Choose A/B/C/D/E/F/G/H to get 2 points, and choose I to get no points.

	H. Temperature and humidity records are available<br>I. None	
	23. Configurations of the experimental equipment comply with the following requirements:<br>A. At least 1 microplate reader or fluorescence analyzer for experimental testing<br>B. At least one plate washing instrument for washing the experimental plate<br>C. At least one oscillator for mixing experimental reagents<br>D. At least one computer (including printers) for data processing<br>E. At least 1 thermostat or water bath for experimental thermostatic treatment<br>F. At least one 2 – 8 °C refrigerator for reagent storage<br>G. At least 2 multichannel samplers for experimental sampling<br>H. At least 2 singlechannel sampler for experimental sampling<br>I. Have Puncher for punching DBS<br>J. At least one ultraclean worktable for experimental operation of bacterial inhibition methods<br>K. Have general low-value laboratory supplies<br>L. None	Choose A/B/C/D/E/F/G/H/I/J/K to get 1 point, and choose L to get no points.
(IV) Rules construction	24. Are there ideal and constantly updated personnel rules?<br>A. YES. There is a personnel position responsibility rule and it is constantly updated.<br>B. YES. There is personnel conduct rule and it is constantly updated.<br>C. None.	Choose A/B to get 2 points, and choose C to get no points.
	25. Are there ideal and constantly updated rules for the diagnosis and treatment of IEMs?<br>A. YES<br>B. NO	Choose A to get 4 points, and choose B to get no points.
	26. Are there ideal and constantly updated rules for case management?<br>A. YES. There is a referral rule and it is constantly updated.<br>B. YES. There is a recall and follow-up rule and it is constantly updated.<br>C. YES. There is a statistical summary and reporting rule and it is constantly updated.<br>D. None	Choose A/B/C to get 1 point, and choose D to get no points.
	27. Are there ideal and constantly updated rules for archives management?<br>A. YES. There is a file management rule for confirmed patients and it is constantly updated.<br>B. YES. There is an information management and security rule for confirmed patients and it is constantly updated.	Choose A/B to get 1 point, and choose C to get no
	28. Are there ideal and constantly updated rules for laboratory equipment and specimen management?<br>A. YES. There is a rule for equipment management.<br>B. There is a rule for reagents and materials management.<br>C. There is a rule for specimen registration and preservation.<br>D. None	Choose A/B/C to get 1 point, and choose D to get no points.
(V) Information system construction	29. Is there a well-established information system for screening data management?<br>A. YES. There is a neonatal screening information system.<br>B. YES. The whole process, from blood collection to reporting, is computerizedt.<br>C. YES. There is an information module for preliminary screening.<br>D. YES. There is an information module for preliminary diagnosis.	Choose A/B/C/D to get 1 point, and choose E to get no points.

E. None

30. Is there a well-established information system for case records, diagnosis and treatment, and follow-up management?

Choose A/B to get 1 points, choose C to get 2 points, and chosse D to get no point.

A. YES. There is an information system for the diagnosis and treatment of inherited metabolic diseases.

B. YES. The entire process, from rescreening (confirmation) and diagnosis to follow-up, is computerized

C. YES. There is a complete information module for recall.

D. None

Screening

(VI) Prescreening health education and publicity

31.As for informed consent, the following contents are included:

Choose A/B/C/D/E/F to get 0.5 point, and choose G to get no points.

A. Name of the mother, neonatal sex, date of birth and medical record number of hospitalization in the informed consent.

B. Sections for popularizing newborn screening health education publicity and related policies.

C. Section for informed choice of the family member of the child, including the signature and date of the signature by the guardian.

D. If the newborn's guardians do not agree to the screening after the neonatal screening health education, the guardians are informed of the possible adverse consequences of disease.

E. If the newborn's guardians do not agree to accept the neonatal screening, the guardian's signature, signature date, current address and contact information are recorded.

F. There is a medical (caregiver) statement section (example statement: I have informed the caregiver of the nature, purpose, risk, necessity and cost of genetic metabolic disease screening, and have answered any questions related to this examination), medical (caregiver) signature, and date of the signature.

G. None

(VII) Pretesting quality control

32. Do the consumable materials for blood collection meet the requirements?

Choose A to get 1 points, and choose B to get no points.

A. YES. The filter paper for the DBS sample making has been approved by the Food and Drug Administration department for registration or filing.

B. NO. The filter paper for the DBS sample making has not been approved by the Food and Drug Administration department for registration or filing.

33. Do the equipment and reagents meet the requirements?

Choose A/B/C/D to get 1 points, and choose E to get no points.

A. YES. Equipment and reagents for NBS have approval registration or filing.
B. YES. There are records for equipment maintaining.
C. YES. There are inbound and outbound records for all reagents.
D. YES. The instrument is calibrated annually.
E. None

34.Are there records for specimens acceptance, including the time of receipt of the specimens, the number of specimens, and the state of the specimens?

Choose A to get 4 points, and choose B to get no points.

A. YES
B. NO

35.As for the specimen collection card information, the following contents are included:

10 points can only be scored if the contents of the 5 information cards are completed; otherwise, 2 points are deducted for each missing item in each information card, until 10 points are deducted.

A. Date of birth of the child
B. Gender of the child
C. Weight of the child
D. Gestational age of the child

	E. Mother's name<br>F. Date of blood collection, blood collector	
	38. The number of the quarterly ☐ or yearly ☐ specimens:___, in which the number of unqualified specimens:___, and the unqualified specimen rate:___.	If the unqualified specimen rate < 0.5%, 10 points are scored. If 0.5%⩽ the unqualified specimen rate <1%, 5 points are scored. If the unqualified specimen rate ⩾ 1%, no point is scored.
	39. The number of the quarterly ☐ or yearly ☐ unqualified specimens:___, in which the number of the specimens recollected within 42 days:___, the recollection rate of unqualified specimens:___.	If the recollection rate of unqualified specimens >90%, 10 points are scored. If 80%⩽ the recollection rate of unqualified specimens ⩽90%, 5 points are scored. If the recollection rate of unqualified specimens < 80%, no points are scored.
	40. The median of the time from the collection of the DBS sample to the receipt of the DBS sample in the laboratory (annual statistics)?<br>A. ⩽5 work days<br>B. >5 work days	Choose A to get 10 points, and choose B to get no points.
	41. The total number of specimens collected during the annual screening:___, the number of specimens transported within 5 working days:___, and the timely rate of blood film turnover before testing:__.	If the rate is > 90%, 16 points are scored. If 80%⩽ the rate ⩽90%, 8 points are scored. If the rate < 80%, no points are scored.
(VIII) Testing quality control	42. As for completeness of the laboratory testing SOP, the following contents are included:<br>A. There are SOPs for the collection, storage and processing of DBS.<br>B. DBS processing meet the SOP.<br>C. There is an SOP for the use of testing technology and/or testing equipment.<br>D. The uso of testing technology and/or testing equipment meet the SOP.<br>E. There is a quality control rule for the collection and making process of DBS.<br>F. There is a quality control rule for the acceptance of DBS samples.<br>G. The biosafety SOP comply with the relevant laboratory biosafety guidelines (refer to "WS/T 442-2014 Clinical Laboratory Biosafety Guidelines").<br>H. None	Choose A/B/C/D/E/F/G to get 2 points, and choose H to get no points.
	43. Is the performance of laboratory measurement systems checked regularly?<br>A. YES. Once a year or more.<br>B. YES. Every two years.<br>C. YES. Every three years.<br>D. Never done.	Choose A/B to get 4 points, and choose C/D to get no points.
	44. Number of testing items which has the internal quality control:___. The frequency of internal quality control for Phe testing:___. The frequency of internal quality control for TSH testing:___. The number of the concentration level in each plate for quality control:___.	If the number of test items is greater than or equal to 2, 3 points are scored, and less than 2 is not scored; As for Phe testing and TSH testing, the quality control frequency of each plate is greater than or equal to 1 time to get 3 points, and the frequency of less than 1 plate is not to be scored; If the quality control level of each plate is greater than or equal to two concentrations, 3 points are scored, and if the quality control level of each plate is less than two concentrations, no point is scored.
	45. Does the internal quality control for PKU and TSH laboratory testing meet the requirements?<br>A. YES. There is quality control chart for internal quality control.<br>B. YES. There are analysis records or reports of the reasons for the loss of internal quality control.<br>C. YES. There are corrective measures after the loss of internal quality control.<br>D. None	Choose A/B/C to get 4 points, and choose D to get no points.
	46. For Phe, are the accumulated CV% in control for at least half a year?<br>A. YES<br>B. NO	Choose A to get 9 points, and choose B to get no
	47. For Phe, are the accumulated CV% not greater than 1/3 of the total allowable error of the external quality assessment?<br>A. YES<br>B. NO	Choose A to get 9 points, and choose B to get no points.
	48. For TSH, are the accumulated CV% in control for at least half a year?<br>A. YES<br>B. NO	Choose A to get 9 points, and choose B to get no

	49. For TSH, are the accumulated CV% not greater than 1/3 of the total allowable error of the external quality assessment?<br>A. YES<br>B. NO	Choose A to get 9 points, and choose B to get no points.
	50. The number of NBS testing items participating in NCCL external quality assessment activities each year:__. The participating frequency of external quality assessment for PKU:__. The participating frequency of external quality assessment for TSH:__.	If the number of items is equal to 2, 10 points are scored, and 10 points are deducted for less than 2 items. 5 points for each item whose participation frequency is greater than or equal to 1 time per year, 5 points are deducted for 0 participation.
	51. As for the EQA passing status of the Phe testing, does the institution obtain a certificate?<br>A. YES<br>B. NO	Choose A to get 10 points, and choose B to deduct 10 points.
	52. As for the EQA passing status of the TSH testing, does the institution obtain a certificate?<br>A. YES<br>B. NO	Choose A to get 10 points, and choose B to deduct 10 points.
(IX) Posttesting quality control	53. The number of the quarterly ☐ or yearly ☐ issued reports:___, in which the number of the reports issued within 5 working days from the date of receiving qualified DBS samples:___, the percentage of reports issued by the laboratory within 5 working days from the date of receiving qualified DBS samples to the total reports:___.	If the rate=100%, 10 points are scored. If 90%≤ the rate < 100%, 5 points are scored. If 80%≤ the rate < 90%, no points are scored. If the rate is < 80%, 5 points are deducted.
	54. The test report should contain the following information:<br>A. The mother's name<br>B. Child's age<br>C. Child's birth date<br>D. Identification number:<br>E. Date of sampling, testing, and report issued:<br>F. Screening testing results:<br>G. Tester and results reviewer's signature:<br>H. The reviewer has intermediate or above technical titles/positions:<br>I. None	Choose A /B/C/D/E/F/G/H to get 2 points, and choose I to get no points.
	55. Which of the following requirements does your laboratory's inspection report release process meet:(Single choice or multiple choice according to the actual situation)<br>A. Quality control measures to reduce errors in the issuance of inspection<br>B. There is SOP document for the quantitative and qualitative judgment of the screening results of neonatal genetic metabolic diseases:<br>C. Do the quantitative and qualitative judgments of the screening results of neonatal genetic and metabolic diseases meet the SOP documents formulated by the institution:<br>D. There is quality control rule for the recall process of NBS positive<br>E. None	Choose A /B/C/D to get 2 points, and choose E to get no points.
	56. The number of notified children with positive PKU screening results in the quarter:__, and the total number of children with positive PKU screening results during the same period:__, notification rate of children with positive PKU testing results:__.	25 points are scored for the notification rate of 100% , and 25 points are deducted for the notification rate less than 100%.
	57. The number of notified children with positive CH screening results in the quarter:__, and the total number of children with positive CH screening results during the same period:__, notification rate of children with positive CH testing results:__.	25 points are scored for the notification rate of 100% , and 25 points are deducted for the notification rate less than 100%.
	58. The number of the quarterly ☐ or yearly ☐ children with positive PKU screening results:__, in which the number of recalled children with positive PKU screening results:__, recall rate of children with positive PKU test results:__.	If the rate is > 90%, 20 points are scored. If 80%≤ the rate < 90%, 10 points are scored. If 70%≤ the rate < 80%, 5 points are scored. If 60%≤ the rate < 70%, 2 points are scored. If the rate is < 60%, no point is scored.

	59. The number of the quarterly □ or yearly □ children with positive PKU screening results: , in which the number of recalled children with positive PKU screening results: , recall rate of children with positive PKU test results: .	If the rate is > 90%, 20 points are scored. If 80%≤ the rate < 90%, 10 points are scored. If 70%≤ the rate < 80%, 5 points are scored. If 60%≤ the rate < 70%, 2 points are scored. If the rate is < 61%, no point is scored.
	59. The number of the quarterly ☐ or yearly ☐ children with positive CH screening results:__, in which the number of recalled children with positive CH screening results:__, recall rate of children with positive CH test results:__.	If the rate is > 90%, 20 points are scored. If 80%≤ the rate < 90%, 10 points are scored. If 70%≤ the rate < 80%, 5 points are scored. If 60%≤ the rate < 70%, 2 points are scored. If the rate is < 60%, no point is scored.
	60. The number of the quarterly ☐ or yearly ☐ recalled children with positive PKU screening results:__, in which the number of confirmed children with positive PKU screening results:__, positive predictive value of PKU screening testing:__.	If positive predictive value is > 0.08, 22 points are scored. If 0.05≤ the value ≤ 0.08, 11 points are scored. If the value is < 0.05, no point is scored.
	61. The number of the quarterly ☐ or yearly ☐ recalled children with positive CH screening results:__, in which the number of confirmed children with positive CH screening results:__, positive predictive value of CH screening testing:__.	If positive predictive value is > 0.08, 22 points are scored. If 0.05≤ the value ≤ 0.08, 11 points are scored. If the value is < 0.05, no point is scored.
	62. The number of the quarterly ☐ or yearly ☐ confirmed PKU patients:__, in which the number of PKU patients with negative screening results:__, false negative rate of PKU screening testing:__.	If the false negative rate is < 0.3, 10 points are scored. If 0.3≤ the rate ≤ 0.8, 5 points are scored. If false negative rate is > 0.8, no point is scored.
	63. The number of the quarterly ☐ or yearly ☐ confirmed CH patients:__, in which the number of CH patients with negative screening results:__, false negative rate of CH screening testing.:__.	If the false negative rate is < 0.3, 10 points are scored. If 0.3≤ the rate ≤ 0.8, 5 points are scored. If false negative rate is > 0.8, no point is scored.
(X) Follow up	64. The number of the follow-ups:__, in which the number of positive follow-ups: , the number of negative follow-ups:__, the positive follow-up rate:__, the negative follow-up rate:__.	If 90% ≤ the rate ≤ 100%, 13 points are scored. If 60% < the rate < 90%, 6 points are scored. If the rate ≤ 60%, no point is scored.
(XI) Preservation of testing files and specimens	65. Does your laboratory save the original data of each test result, including standard curves, quality control results, screening results, etc.:<br>A. YES<br>B. NO	Choose A to get 2 points, and choose B to get no points.
	66. Does your laboratory keep relevant quality control data, including indoor quality control charts, feedback of laboratory quality evaluation results, reasons for loss of control, corrective measures, etc.:<br>A. YES<br>B. NO	Choose A to get 2 points, and choose B to get no points.
	67. The storage temperature of specimens in your laboratory is:<br>A. ＜0°C<br>B. 2-8°C<br>C. 25°C<br>D. Room temperature	Choose A/B to get 2 points, and choose C/D to get no points.
**Diagnosis and treatment**		
(XII) Case	68. Do all positive screening tests have a clear confirmed diagnosis?<br>A. YES<br>B. NO	Choose A to get 2 points, and choose B to get no
	69. As for the case writing, the following contents are included:<br>A. The date of assessment of the screening results.<br>B. The date of diagnosis/case treatment.<br>C. The date of treatment/intervention (if feasible).<br>D. The confirmed results.<br>E. The treatment results of the final case (Intervention, no intervention, follow-up disappearance).	Choose A/B/C/D to get 1 point, and choose D to get 2 points.

	70. The proportion of the number of standardized diagnoses of PKU in the number of children with positive PKU screening results: __. The calculation formula is as follows: PKU standard diagnosis number/ the number of children with positive PKU screening results x 100%.	If the proportion=100%, 30 points are scored. If 90%≤ the proportion ≤ 100%, 20 points are scored. If 80%≤ the proportion ≤90%, 10 points are scored. If 50%≤ the proportion ≤80%, no points are scored. If the proportion＜50%, 5 points are deducted.
	71. The proportion of the number of PKU patients who were screened and diagnosed during the neonatal period (28 days after birth) in the number of children with PKU:__. The calculation formula is as follows: the number of children with PKU diagnosed in the neonatal period/the number of children with diagnosed PKU x 100%.	If the proportion=100%, 30 points are scored. If 90%≤ the proportion ≤ 100%, 20 points are scored. If 80%≤ the proportion ≤90%, 10 points are scored. If 50%≤ the proportion ≤80%, no points are scored. If the proportion＜50%, 5 points are deducted.
	72. The proportion of the number of standardized diagnoses of CH in the number of children with positive CH screening results:__. The calculation formula is as follows: CH standard diagnosis number/ the number of children with positive CH screening results x 100%.	If the proportion=100%, 30 points are scored. If 90%≤ the proportion ≤ 100%, 20 points are scored. If 80%≤ the proportion ≤90%, 10 points are scored. If 50%≤ the proportion ≤80%, no points are scored. If the proportion＜50%, 5 points are deducted.
	73. The proportion of the number of CH patients who were screened and diagnosed during the neonatal period (28 days after birth) in the number of children with CH:__. The calculation formula is as follows: the number of children with CH diagnosed in the neonatal period/the number of children with diagnosed CH x 100%.	If the proportion=100%, 30 points are scored. If 90%≤ the proportion ≤ 100%, 20 points are scored. If 80%≤ the proportion ≤90%, 10 points are scored. If 50%≤ the proportion ≤80%, no points are scored. If the proportion＜50%, 5 points are deducted.
(XIII) Treatment and follow-up	74. The number of children with PKU who were treated according to the "Technical Specifications for Newborn Disease Screening" accounted for the number of children diagnosed with PKU:__. The calculation formula is as follows: the number of children with PKU treated in accordance with the "Technical Specifications for Newborn Disease Screening"/the number of children diagnosed with PKU x 100%.	If the proportion=100%, 30 points are scored. If 90%≤ the proportion ≤ 100%, 20 points are scored. If 80%≤ the proportion ≤90%, 10 points are scored. If 50%≤ the proportion ≤80%, no points are scored. If the proportion＜50%, 5 points are deducted.
	75. The monitoring period of Phe concentration in children with PKU was (Multi-choice):<br>A. 1-2 weeks<br>B. 1 month<br>C. 3 months<br>D. 6 months<br>E. 12 months	Choose A to get 10 points, choose B to get 8 points, choose C to get 6 points, choose D to get 4 points, and choose E to get 2 points.
	76. The proportion of PKU patients with normal physical development status:__. The calculation formula is as follows: the number of children with PKU with normal physical development at the ages of 3 and 6/the total number of treated children with PKU.	If the proportion=100%, 60 points are scored. If 90%≤ the proportion ≤ 100%, 40 points are scored. If 80%≤ the proportion ≤90%, 30 points are scored. If 50%≤ the proportion ≤80%, no points are scored. If the proportion＜50%, 10 points are deducted.

	77. The proportion of PKU patients with normal intelligent development status: . The calculation formula is as follows: the number of children with PKU with normal intelligent development at the ages of 3 and 6/the total number of treated children with PKU.	If the proportion=100%, 60 points are scored. If 90%≤ the proportion ≤ 100%, 40 points are scored. If 80%≤ the proportion ≤90%, 30 points are scored. If 50%≤ the proportion ≤80%, no points are scored. If the proportion＜50%, 10 points are deducted.
	78. The number of children with CH who were treated according to the "Technical Specifications for Newborn Disease Screening" accounted for the number of children diagnosed with CH:__. The calculation formula is as follows: the number of children with CH treated in accordance with the "Technical Specifications for Newborn Disease Screening"/the number of children diagnosed with CH x 100%.	If the proportion=100%, 30 points are scored. If 90%≤ the proportion ≤ 100%, 20 points are scored. If 80%≤ the proportion ≤90%, 10 points are scored. If 50%≤ the proportion ≤80%, no points are scored. If the proportion＜50%, 5 points are deducted.
	79. The monitoring period of FT4/TSH concentration in children with CH was (Multi-choice):<br>A. 1-2 weeks<br>B. 1 month<br>C. 3 months<br>D. 6 months<br>E. 12 months	Choose A to get 10 points, choose B to get 8 points, choose C to get 6 points, choose D to get 4 points, and choose E to get 2 points.
	80. The proportion of CH patients with normal physical development status:__. The calculation formula is as follows: the number of children with CH with normal physical development at the ages of 3 and 6/the total number of treated children with CH.	If the proportion=100%, 60 points are scored. If 90%≤ the proportion ≤ 100%, 40 points are scored. If 80%≤ the proportion ≤90%, 30 points are scored. If 50%≤ the proportion ≤80%, no points are scored. If the proportion＜50%, 10 points are deducted.
	81. The proportion of CH patients with normal intelligent development status:__. The calculation formula is as follows: the number of children with CH with normal intelligent development at the ages of 3 and 6/the total number of treated children with CH.	If the proportion=100%, 60 points are scored. If 90%≤ the proportion ≤ 100%, 40 points are scored. If 80%≤ the proportion ≤90%, 30 points are scored. If 50%≤ the proportion ≤80%, no points are scored. If the proportion＜50%, 10 points are deducted.
(XIV) Medical record management	82. Whether the PKU specialist archives and management rules are established and the medical records of children with PKU are established and properly managed?<br>A. YES<br>B. NO	Choose A to get 10 points, and choose B to get no points.
	83. Whether he CH specialist archives and management rules are established and the medical records of children with CH are established and properly managed?<br>A. YES<br>B. NO	Choose A to get 10 points, and choose B to get no points.

**For blood sample collection agencies**	**Questionnaires**	**Scoring criteria**
(I) Personnel requirements	84.Do you have a secondary specialized school degeree or above related to medicine and have been engaged in medical clinical work for more than 2 years?<br>A. YES<br>B. NO	At least one blood collection staff meets the requirements to get 5 points, otherwise, no point is scored.

	85. How many people have been engaged in blood collection and received at least one Newborn Screening training in your institution :<br>A. <5<br>B. 5-10<br>C.>10	All the personnel engaged in the Newborn Screening blood collection received at least one Newborn Screening training and obtained the training certificate to get 10 points, otherwise socre 0 point.
(II) Institution construction requirements	86.Provide employees with qualified and applicable protective equipment and equipment, including ethanol, gloves, disposable lancets, etc.<br>A. YES<br>B. NO	Choose A to get 5 points, and choose B to get no points.
	87. The handling and reporting mechanism for occupational exposure.<br>A. YES<br>B. NO	Choose A to get 5 points, and choose B to get no
	88. Annually participate in the quality control from the NBS centers.<br>A. YES<br>B. NO	Choose A to get 10 points, and choose B to get no
	89. Collect and analyze relevant data of NBS.<br>A. YES<br>B. NO	Choose A to get 5 points, and choose B to get no
	90. Timely report relevant data of NBS.<br>A. YES<br>B. NO	Choose A to get 5 points, and choose B to get no
(III) Publicity and health education	91. Which of the following specifications does your organization meet for educational propaganda and informed consent ? (single or multiple selection according to the actual situation)<br>A. Disseminating to families of newborn babies the policy of screening for neonatal genetic metabolic diseases and the identification of simple medical knowledge<br>B. The blood smear collectors truthfully inform the guardians of newborns about the purpose and significance of neonatal genetic metabolic disease screening before implementing blood smear collection<br>C. The signature and date of the guardian when the family members of the child were screened after being informed<br>D. If the guardian does not agree to accept the new screening, whether or not the guardian signs, the date of signature, the present address of the guardian and the means of contact are recorded<br>E. After the newborn screening health education informs the guardian of the child, if the guardian does not agree to the screening, whether to inform the adverse consequences caused by the disease<br>F. In the statement of medical (nursing) personnel, there is the signature and date of signature of medical (nursing) personnel	Multiple-choice questions : 20 points for A, 3 points for B, C, D, E, and 35 points for full.
(IV) Blood collection	92. Which of the following specifications does your institution's specimen collection meet ? (single or multiple selection according to the actual situation)<br>A. Operate according to sterile routine, and the number is unique<br>B. Heel blood was collected according to the steps of blood tablet collection for neonatal genetic metabolic disease screening, and filter paper dried blood tablets were made and dried in accordance with the specifications<br>C. Blood collection is performed between 72 hours and 7 days after birth	Multiple-choice questions : 10 points for A, 20 points for B, 30 points for C, and 60 points for full.
	93. Which of the following specifications does the specimen preservation of your institution meet ? (single or multiple selection according to the actual situation)	Multiple-choice questions : 5 points for A, 10 points for B, 5 points for C and 20 points for full.

	A. Put the qualified filter paper dried blood tablets in A sealed bag in time and store them in A refrigerator at 2 ~ 8℃, or below 0℃ if necessary<br><br>B. All blood slides shall be treated in accordance with specimens of blood-borne infectious diseases, and specimens of special infectious diseases such as AIDS shall be marked and packaged separately<br><br>C. Sealing of Newborn Screening specimens	
	94.Whether the specimens collected by your organization are delivered within 5 days?<br>A. YES<br>B. NO	Choose A to get 30 points, and choose B to get no points.
	95. Whether there is a specimen transfer registration form within your institution ?<br>A. Part<br>B. All<br>C. No	Choose B to get 10 points, and choose A or C to get no points.
	96. Does your organization have re-collection records for abnormal / invalid samples ?<br>A. Part<br>B. All<br>C. No	Choosing B to get 10 points, and choose A or C to get 0 points.
(V) Quality of specimens	97. Unqualified specimens collected by your institution in 2020:	Unqualified blood slide rate < 0.5 %, 40 points. 0.5 % ≤ unqualified blood slide rate < 1 %, 5 points. Unqualified blood slide rate ≥ 1 %, 0 points.
	98. The proportion of blood collection institutions reaching the Newborn Screening center within an average of five working days specimens in 2020:	The proportion of timely delivery of blood slides was 100 %, and the score was 40. Score for timely delivery proportion of blood slides * 40 points.
(VI) Archives preservation	99. What are the following normative requirements for the preservation of blood collection files in your institution ? (single or multiple selection according to the actual situation)<br><br>A. Complete registration and archiving of information, including information on live births, screening, and preservation for at least 10 years<br><br>B. Procedures for preservation and backup of paper archives or electronic archives and information materials	Multiple-choice questions : 8 points for A, 2 points for B, and 10 points for full.
